# Studies on improving semen quality and increasing pregnancy chances through the in vitro addition of L-carnitine and coenzyme Q10 to semen in patients with asthenozoospermia

**DOI:** 10.1186/s12610-022-00167-7

**Published:** 2022-10-04

**Authors:** Chengren Gou, Zidong Zhou, Zongping Chen, Kun Wang, Congcong Chen, Bo Chen, Ningrui Pan, Xu He

**Affiliations:** 1 Department of Urology, Affiliated Hospital of Zunyi Medical University, Zunyi, 563000 Guizhou China; 2 Department of Urology, People’s Hospital of Guizhou Province, Guiyang, 550002 Guizhou China

**Keywords:** Asthenozoospermia, Male infertility, Pregnancy rate, Prodom, L-carnitine, Coenzyme Q10, Asthénozoospermie, Infertilité masculine, Taux de grossesse, Prodom™, L-carnitine, Coenzyme Q10

## Abstract

**Background:**

At present, L-carnitine (LC) and coenzyme Q10 (CoQ10), as used clinically to treat male infertility caused by asthenozoospermia (ASZ) is still mainly administered orally, but some patients with ASZ still show no significant improvement in sperm motility and spouse pregnancy rate. Prodom is a device used to assist reproduction, which is temporarily fitted onto the penis to facilitate conception by helping the wife inject a certain drug into the vagina. This study used Prodom-assisted LC/CoQ10 in the treatment of patients with ASZ and evaluated the effect of this method on sperm motility and clinical pregnancy, with the goal of finding a comfortable, low-cost, effective method.

**Results:**

During the trial period, 232 cases completed the trial, while 25 cases did not. During in vitro testing, the progressive sperm motility in the LC group, CoQ10 group, LC combined with CoQ10 group, and the semen blank control group was 24.3 ± 4.6% and 38.1 ± 5.1%, 23.0 ± 4.8% and 36.9 ± 4.4%, 28.4 ± 5.0% and 43.8 ± 5.4%, 19.7 ± 4.4% and 26.0 ± 4.9%, respectively. There were statistically significant differences in progressive sperm motility among the groups (all *P* values < 0.05). The pregnancy rates of the Prodom-assisted LC treatment group, Prodom-assisted CoQ10 treatment group, Prodom-assisted LC combined with CoQ10 treatment group, and oral LC combined with CoQ10 treatment group in the clinical treatment stage were 38.2, 35.4, 57.1, and 30.3%, respectively; the time to conception was 6.1 ± 1.8, 6.2 ± 1.8, 3.4 ± 0.9, and 7.9 ± 2.0, months respectively; and the treatment costs were $2350 ± 457, $2455 ± 434, $1348 ± 411, and $2684 ± 334, respectively. The differences in pregnancy rate, time to conception, and treatment costs among the groups were statistically significant (all *P* values < 0.05).

**Conclusions:**

The supplementation of in vitro semen with LC/CoQ10 can improve sperm motility. LC/CoQ10 injected into the spouse’s vagina with the assistance of a Prodom can increase the pregnancy rate, shorten the time to conception, and reduce the cost of treatment in patients with ASZ.

**Trial registration:**

ChiCTR2000040349 (registry: http://www.chictr.org.cn/).

Date of registration: November 28, 2020.

## Résumé

**Contexte:**

À l’heure actuelle, la L-carnitine (LC) et le coenzyme Q10 (CoQ10), tels qu’utilisés en clinique pour traiter l’infertilité masculine due à une asthénozoospermie (AZS), sont encore principalement administrés par voie orale, mais certains patients atteints d’AZS ne montrent pas toujours d’amélioration significative de la motilité des spermatozoïdes, ni de grossesse chez la conjointe. Prodom™ est. un dispositif utilisé pour aider à la reproduction, qui est. temporairement installé sur le pénis pour faciliter la conception en aidant la femme à injecter certains médicaments dans le vagin. Cette étude a utilisé une association CL + CoQ10 assistée par Prodom™ pour traiter des patients atteints d’AZS et a évalué l’effet de cette méthode sur la mobilité des spermatozoïdes et la grossesse clinique, dans le but de trouver une méthode confortable, peu coûteuse et efficace.

**Résultats:**

Au cours de cet essai thérapeutique, 232 cas sont allés au bout de l’étude, alors que 25 se sont arrêtés. Au cours des tests in vitro, la mobilité progressive des spermatozoïdes dans le groupe LC, le groupe CoQ10, le groupe CL + CoQ10 et dans le groupe témoin de sperme, était respectivement de 24,3 ± 4,6% et 38,1 ± 5,1%, de 23,0 ± 4,8% et 36,9 ± 4,4%, de 28,4 ± 5,0% et 43,8 ± 5,4%, et de 19,7 ± 4,4% et 26,0 ± 4,9%. La mobilité progressive des spermatozoïdes était significativement différentes entre les groupes (toutes les valeurs de *P* < 0,05). Les taux de grossesse dans le groupe traité par LC assistée par Prodom™, dans le groupe traité par CoQ10 assisté par Prodom™, dans le groupe traité par une association CL + CoQ10 assistée par Prodom™, et dans le groupe traité cliniquement par CL par voie orale associée au CoQ10, étaient respectivement de 38,2%, 35,4%, 57,1% et 30,3%; le délai de conception était respectivement de 6,1 ± 1,8, 6,2 ± 1,8, 3,4 ± 0,9 et 7,9 ± 2,0, mois; et les coûts de traitement étaient respectivement de 2350 ± 457 $, 2455 ± 434 $, 1348 ± 411 $ et 2684 ± 334 $. Les différences dans le taux de grossesse, le délai de conception et les coûts de traitement entre les groupes étaient statistiquement significatives (toutes les valeurs de *P* < 0,05).

**Conclusions:**

La supplémentation en LC/CoQ10 du sperme in vitro peut améliorer la mobilité des spermatozoïdes. L’association LC + CoQ10 injectée dans le vagin de la conjointe avec l’aide d’un Prodom™ peut augmenter le taux de grossesse, raccourcir le délai de conception et réduire le coût du traitement chez les patientes atteints d’AZS.

Numéro d’enregistrement de l’essai ChiCTR2000040349 (registre: http://www.chictr.org.cn/).

Date d’enregistrement: 28 novembre 2020.

## Introduction

Asthenozoospermia (ASZ), which is a condition indicated by a semen sample with reduced sperm motility, is considered one of the main factors contributing to male infertility [1]. According to reports, ASZ accounts for 19–20% of male infertility [2, 3]. Studies have shown that when a sperm’s energy supply is low, excessive reactive oxygen species produced by white blood cells, germ cells, and abnormal spermatozoa can cause damage to cell DNA, lipids and proteins. This affects the integrity of the sperm plasma membrane and sperm viability, which is related to the occurrence of ASZ [4–7].

L-carnitine (LC) is a water-soluble antioxidant widely found in the male reproductive tract, especially in the epididymis. It transports long-chain fatty acids to mitochondria for β-oxidation, promoting oxidative reactions and providing energy for spermatozoa [8, 9]. Coenzyme Q10 (CoQ10), a fat-soluble antioxidant, is involved in electron transport during mitochondrial oxidative phosphorylation and protects cells from attack by free oxygen. CoQ10 is a key component of the mitochondrial respiratory chain and plays an important role in maintaining energy production, cell membranes and lipoprotein metabolism as a lipid-soluble chain breaking antioxidant [10, 11]. Studies have found that the levels of CoQ10 in spermatozoa of ASZ patients are significantly reduced [11–13], and the concentration of endogenous LC in spermatozoa is significantly positively correlated with their quality [14]. With the decrease in LC and CoQ10 concentrations in vivo, sperm mitochondrial function is reduced, and the metabolic rate of epididymal spermatozoa is also decreased, leading to male infertility [7, 8, 15]. In addition, in clinical practice, oral treatment of LC and CoQ10 has achieved certain efficacy in improving semen quality, such as sperm survival rate and progressive sperm motility [7, 8, 16, 17].

However, because of the complex metabolic mechanisms of the human body, oral treatment with LC and CoQ10 cannot completely improve ASZ through in vivo approaches [17–20]. Therefore, to achieve a more ideal therapeutic effect, the focus of treatment has shifted to improving ASZ through in vitro approaches [21, 22].

The author has used chymotrypsin and urokinase to treat male infertility caused by impaired semen liquefaction through a special device designed by us, namely, a Prodom, and the results were encouraging [23, 24]. The aforementioned Prodom is an assisted reproductive device that is temporarily fitted onto the penis to facilitate the husband and wife injecting a certain drug into the vagina simultaneously with ejaculation during coitus. Once ejaculation occurs, the drug is inserted in the vagina and mixed with semen to improve the semen composition, enhance sperm motility and survival rate, and contribute to conception. There are currently no reports in clinical practice of Prodom-assisted LC and CoQ10 therapy for ASZ. Therefore, the purpose of this study is to examine the ability of the Prodom to aid in the delivery of LC and CoQ10 in the treatment of ASZ-induced male infertility and observe its clinical efficacy.

## Materials and methods

### Patients

The current study was a randomized controlled trial. A consecutive series of data covering 257 ASZ patients from November 2020 to December 2021 was collected from the Affiliated Hospital of Zunyi Medical University in China. Inclusion criteria were as follows: (1) Progressive sperm motility less than 32% or sperm motility less than 40% or both; (2) Clinical records of all ASZ patients (outpatient during December 1, 2020 to December 31, 2021) should be complete and accurate, with follow-up visits lasting for 3–12 months. The exclusion criteria were as follows: (1) Male accessory gland inflammation; (2) Leukocytospermia; (3) Papillomavirus infection; (4) Semen hyperviscosity; (5) Testicular volume < 12 ml; (6) Hormonal alterations; (7) Altered accessory gland secretory function (seminal plasma zinc < 2.4umol, fructose <13umol and neutral α-glucosidase < 20 mU an ejaculation.); (8) Absolute ASZ (100% immotile spermatozoa in the ejaculate); (9) Infertile or > 40 years old spouse; (10) Incomplete clinical records; or (11) Willing termination of the treatment or refusal of follow-up visits.

All patients underwent a thorough history-taking and physical examination, including testicular volume assessment. Color ultrasound Doppler was used to measure the size of the testicle, and testicular volume was calculated manually, and according to the formula, testicular volume = length × width × thickness ×π/6. Moreover, an endocrine profile (serum testosterone levels, follicle stimulating hormone, luteinizing hormone and estradiol) and genetic test results (karyotype testing) were obtained. All patients underwent an ultrasound examination of the urogenital tract and testes and routine urine tests.

### Experimental group and intervention measures for each group

**Fig. 1:**
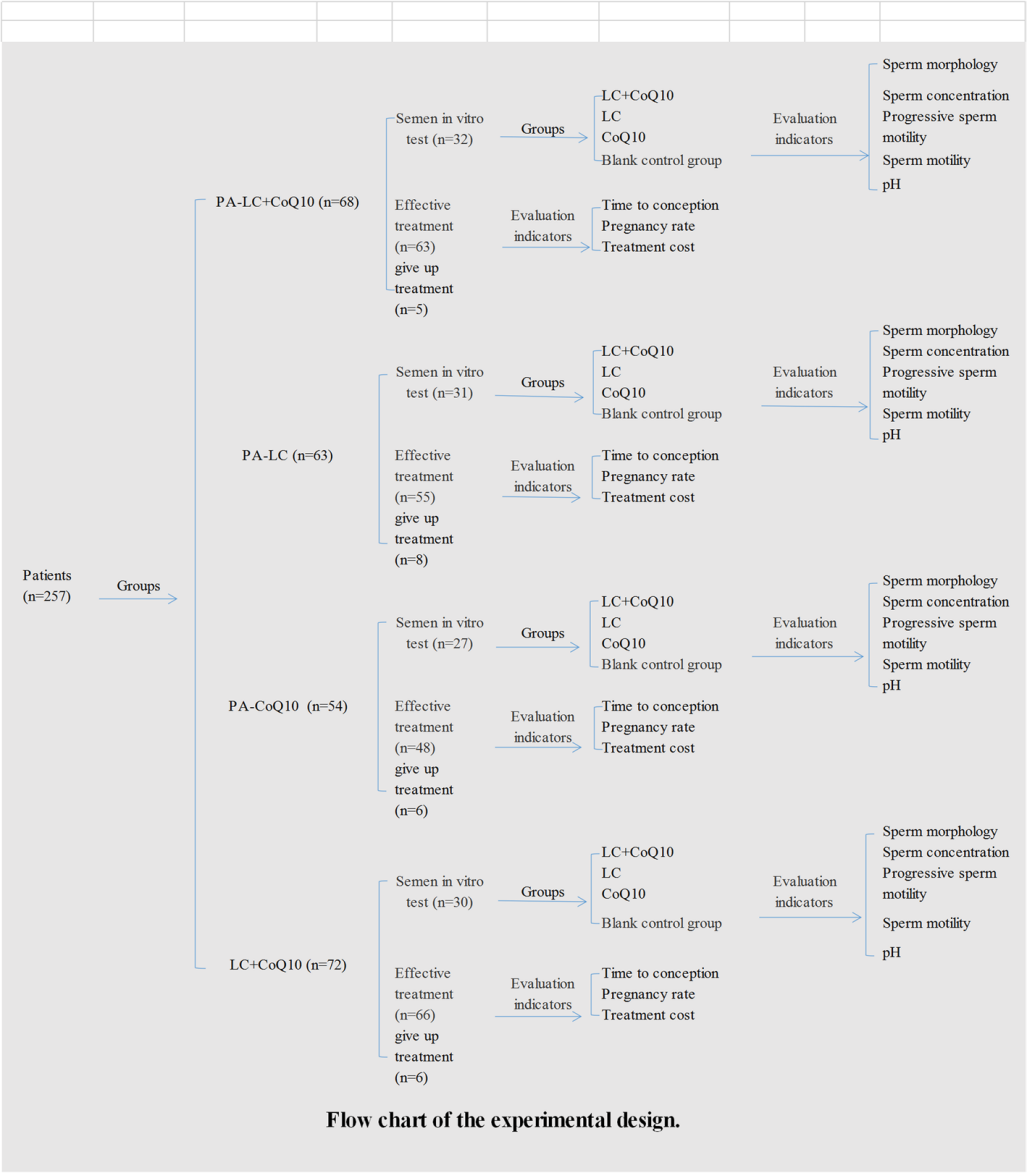
Flow chart of the experimental design. Abbreviations: PA-LC, prodom-assisted L-carnitine treatment; PA-CoQ10, prodom-assisted Coenzyme Q10 treatment; PA-LC + CoQ10, prodom-assisted L-carnitine combined with Coenzyme Q10 treatment; OR-LC + CoQ10, oral L-carnitine combined with Coenzyme Q10 treatment; pH, acidity index

All patients with ASZ were enrolled in the trial that were randomly assigned to the following four groups (see Fig. 1): Prodom-assisted LC (PA-LC) treatment group (*n* = 63), Prodom-assisted CoQ10 (PA-CoQ10) treatment group (*n* = 54), Prodom-assisted LC combined with CoQ10 (PA-LC + CoQ10) treatment group (*n* = 68), and oral LC combined with CoQ10 (OR-LC + CoQ10) treatment group (*n* = 72). Randomization included assigning a number to each patient using a random number table according to the order of visit. The number was then divided by 4, and patients with remainders of 0, 1, 2, and 3 were assigned to the PA-LC, PA-CoQ10, PA-LC + CoQ10, and OR-LC + CoQ10 groups, respectively. Envelope concealment was used to determine which treatment a patient would receive, in which random groups were placed in sequentially coded, sealed, opaque envelopes that were opened by a physician when a qualified subject agreed to participate in the clinical trial.

All patients included in the four groups, before treatment, 120 were randomly selected for semen testing. Through the masturbation method to obtain semen and after natural liquefaction, every semen was divided into four equal parts, and LC, CoQ10, LC joint CoQ10, and Hank’s balance fluid were added to each part. They were divided into the LC addition group, CoQ10 addition group, LC + CoQ10 addition group and blank control group. Randomization included assigning a number to each patient using the random number table according to the order of visit. The number was then divided by 4, and patients with remainders of 0, 1, 2, and 3 were assigned to the LC + CoQ10 in vitro semen addition group, LC in vitro semen addition group, CoQ10 in vitro semen addition group, and blank control group, respectively. The allocation was concealed in sealed envelopes to randomize the distribution. The LC + CoQ10 insemen addition group consisted of a quarter ofthe semen sample, 1 ml LC (concentration: 8 mmol/l, diluted with Hank’s) and 1 ml CoQ10 (concentration: 4 mmol/l, diluted in Hank’s). The LC in addition group consisted of a quarter part of semen and 2 ml LC (concentration: 8 mmol/l, diluted in Hank’s). The CoQ10 in semen addition group consisted of a quarter part of semen and 2 ml CoQ10 (concentration: 4 mmol/l, diluted in Hank’s). The blank control group consisted of a quarter part of semen and 2 ml Hank’s. Each semen sample was incubated at a conventional oxygen concentration in a 37 °C tank of water for 60 minutes, and then sperm motility was assessed.

### The drug, dosage and method of use in this trial

The following drugs were used in this trial:

1. LC reagent, produced by Beijing Soleibao Technology Co., Ltd. China. 5 mg/ bottle, purity 99%. Diluted with Hank’s, to 8 mmol/l preparation [25] (See reference [25] for details).
2. CoQ10 reagent, produced by Beijing Soleibao. 20 mg/bottle, purity 98%. Diluted with Hank’s to 4 mmol/l preparation [25] (See reference [25] for details).
3. LC injection, produced by Changzhou Lanling Pharmaceutical Co., Ltd. China. 5 ml:1 g/bottle. 1 ml injected into the vagina of the spouse through the prodom during intercourse and synchronously with ejaculation.
4. CoQ10 injection, produced by Qingdao Haihui Biochemical Pharmaceutical Co., Ltd. China. 2 ml: 5 mg/bottle. 0.5 ml injected into the vagina of the spouse through the prodom during intercourse and synchronously with ejaculation.
5. LC oral liquid, produced by Northeast Pharmaceutical Group Shenyang First Pharmaceutical Co., Ltd. Dosage, 10 ml:1 g/bottle. 20 ml, twice a day for 3–9 months, orally.
6. CoQ10 oral capsules, produced by Shanghai Xudong Haipu Pharmaceutical Co., Ltd. Dosage, 20 mg/tablet. 20 mg, twice a day for 3–9 months, orally.

### The definition of prodom, its composition, and its operational process

Definition of Prodom: a Prodom is an auxiliary reproductive device that is temporarily fitted onto the penis to aid the husband and wife in injecting a certain drug into the vagina simultaneously with ejaculation during coitus, so that the drug can be well mixed with semen in the vagina, to modify semen composition, enhance sperm motility and survival rate, and contribute to conception.

Composition of Prodom: the Prodom described in this study was mainly composed of polyurethane film (PU film) and an injection catheter. It was coated with pressure-sensitive adhesive on the inner side of the PU film (see references [23, 24] for details).

Operating process of Prodom: (1) Before sex between the husband and wife, drugs used in the study, such as LC and CoQ10, were drawn into a syringe, while the Prodom was pasted onto the erect penis with the pressure-sensitive adhesive set of PU film; (2) The drug from a syringe drawn beforehand was injected into the partner’s vagina. It was synchronized with ejaculation, and the drug blends with spermatozoa in the vagina (see references [23, 24] for details).

### Main monitoring instrument

The semen parameter measurement instruments include an automatic sperm quality analyzer (BEION S3–3, BEIONMED®, Shanghai, China), Elecsys 2010, MODULAR ANALYTICS E170/cobase analyer (Roche, Switzerland), Testsiplets staining slides (Waldeck GmbH & Co. KG, Waldeck, Germany), and an Olympus Optical Microscope (Olympus, Tokyo, Japan).

### Blood specimen collection, hormonal parameter monitoring and genetic testing

All blood specimens were obtained from the subjects in the morning after an overnight fast. Serum testosterone, follicle stimulating hormone, luteinizing hormone, estradiol and prolactin were determined by enzyme-linked immunosorbent assays (ELISA). Genetic testing was performed by G banding karyotype analysis of peripheral blood chromosomes. The above parameters were detected in the clinical laboratory of the Affiliated Hospital of Zunyi Medical University, and Elecsys ELISA kits were used.

### Semen analyses

After a period of 2–7 abstinence days, subjects were asked to collect the ejaculated semen samples after masturbation. Semen parameters (including volume, pH, liquefaction time, sperm count, total sperm number and sperm density, motility, live rate, and morphology) were assessed for each sample. And semen parameters were measured by semiautomatic semen analyzer (BEION S3–3, BEIONMED®, Shanghai, China). The techniques used for semen analyses are performed in accordance with the requirements of the fifth edition of the *WHO laboratory manual for the examination and processing of human semen* in 2010 [1].

### Assessment of pregnancy, miscarriage and the time to conception

Pregnancy was confirmed by any 2 of the following methods: telephone interviews, pregnancy tests, clinical examination, or a birth certificate. Miscarriage was determined on the basis of menopausal history. A positive test result for early pregnancy test confirms that the spouse was pregnant, but the pregnancy terminated spontaneously before 28 weeks and a re-examination of the ultrasound showed that the uterine sac had disappeared. The time to conception was calculated from the beginning of the trial study treatment to the time when pregnancy was confirmed, which is called the time to conception.

### Clinical data

Available data included clinical check-up, semen parameters (including abstinence time, semen volume, pH, liquefaction time, sperm concentration, total sperm number, progressive sperm motility, sperm motility, and sperm morphology), sex hormone detection indicators (including serum testosterone, follicle stimulating hormone, luteinizing hormone, estradiol and prolactin), duration of infertility, routine urine test (urine leukocytes and urine red blood cells), history of ligation of internal spermatic vein, testicular volume, prostatic enlargement, history of chronic prostatitis, history of seminal vesiculitis, history of hematospermia, patient age and spouse (female) age, genetic and family histories, karyotype testing, pregnancy (including live birth, miscarriage), time to conception, and treatment costs (including examination fees, diagnostic fees, the cost of buying drugs and the cost of buying the devices of Prodom). Subjects were evaluated according to the different treatment methods.

### Diagnosis standards

Semen sample with reduced sperm motility are indicative of ASZ. ASZ refers to a progressive sperm motility of less than 32% or sperm motility of less than 40% or both. It was diagnosed using the Fifth Edition Standards in the 2010 *WHO laboratory manual for the examination and processing of human semen* [1].

### Follow-up

The median follow-up time was 9.5 (range 3–12) months. All patients were followed up via telephone and regular outpatient visits. Follow-up items through telephone consultations included health status, pregnancy status, and time to conception. Outpatient follow-ups included general physical examinations, routine blood and blood biochemical examinations, and semen quality analysis.

### Statistical analyses

Statistical analyses were performed using Statistical Package for the Social Sciences (SPSS Inc., Chicago, USA) version 18.0 for Windows (SPSS Inc., Chicago, IL, USA). Selected characteristics (including the clinical data parameters described above that were collected) were compared between treatment group cases using the chi-squared test for categorical variables and *t test* and one-way variance analysis for quantitative data. Numerical variables are presented as the mean ± standard deviation or as the median (minimum, maximum), and categorical variables are presented as percentages. *P* < 0.05 was considered significant.

## Results

### Baseline characteristics of ASZ patients in the PA-LC + CoQ10, PA-LC, PA-CoQ10 and OR-LC + CoQ10 groups

A total of 257 patients, including 232 patients who completed treatment, and 25 patients who stopped treatment (data not shown), were diagnosed with ASZ and registered for the study. As shown in Table 1, there were statistically significant differences (*P* = 0.003) in initial semen volume between the PA-LC + CoQ10, PA-LC, PA-CoQ10 and OR-LC + CoQ10 groups, and the initial semen volume in PA-LC + CoQ10 group was higher than that in other groups; however, there were no statistically significant differences in the remaining parameters among the groups (all *P* > 0.05) for the initial (recruitment) values.

**Table 1:** Baseline characteristics of clinical data of asthenozoospermia in each group

Groups Parameters	PA-LC + CoQ10 (*n* = 63)	PA-LC (*n* = 55)	PA-CoQ10 (*n* = 48)	OR-LC + CoQ10 (*n* = 66)	*P*-value
Patient (Male) Age (Year)	31 (22, 46)	30 (23, 47)	30 (21, 46)	30 (22, 46)	0.116†
Spouse (female) Age (Year)	30 (21, 37)	29 (23, 40)	29 (21, 40)	29 (21, 39)	0.203†
Semen parameters					
Abstinence time (Day)	3 (2, 5)	3 (2, 7)	3 (2, 7)	3 (2, 7)	0.129†
Semen volume (ml)	4.2 ± 0.9	4.0 ± 0.8	3.8 ± 0.8	3.7 ± 0.8	**0.003**†
pH	7.40 ± 0.2	7.4 ± 0.2	7.4 ± 0.2	7.4 ± 0.2	0.563†
Liquefaction time (Min)	15 (10,20)	15 (10,15)	15 (10,20)	15 (10,20)	0.209†
Sperm concentration (10^6^/ml)	46.6 ± 10.7	45.7 ± 11.3	47.1 ± 11.2	47.0 ± 12.0	0.912†
Total sperm number (10^6^)	193.7 ± 56.5	182.3 ± 59.8	179.1 ± 58.8	171.4 ± 52.2	0.166†
Progressive spermmotility, %	21.4 ± 6.4	20.8 ± 4.3	21.5 ± 3.0	21.4 ± 5.2	0.572†
Total sperm motility, %	37.9 ± 6.5	37.4 ± 4.6	36.0 ± 3.3	37.5 ± 5.5	0.695†
Normal sperm morphology, %	9 (6, 12)	8 (6, 12)	9 (7, 12)	8 (6, 12)	0.914†
Length of infertility (Year)	3(1, 6)	2(1, 5)	2(1, 3)	3(1, 5)	0.251†
Testosterone (nmol/l)	16.2 ± 1.2	15.6 ± 1.7	16.0 ± 1.2	15.7 ± 1.6	0.084†
Follicle stimulating hormone (mIU/ml)	3.9 ± 1.1	4.0 ± 1.7	4.0 ± 1.3	3.8 ± 1.6	0.881†
Luteinizing hormone (mIU/ml)	3.8 ± 1.0	3.6 ± 0.7	3.7 ± 0.6	3.8 ± 0.6	0.572†
Estradiol (pmol/l)	74.7 ± 23.8	67.5 ± 19.9	71.0 ± 22.3	70.8 ± 22.5	0.373†
Prolactin (mIU/l)	124.3 ± 13.6	121.7 ± 15.3	121.0 ± 19.6	123.1 ± 18.4	0.733†
Karyotype					
Normal, %	100 (63/63)	100 (55/55)	100 (48/48)	100 (66/66)	*NS*
Routine urine test					
WBC, %					
(+)	4.8 (3/63)	3.6 (2/55)	4.2 (2/48)	3.0 (2/66)	0.652*
(−)	95.2 (60/63)	96.4 (53/55)	95.8 (46/48)	97.0 (64/66)	
Ligation of internal spermatic vein, %					
Yes	14.3 (9/63)	12.7 (7/55)	10.4 (5/48)	7.6 (5/66)	0.943*
No	85.7 (54/63)	87.3 (48/55)	89.6 (43/48)	92.4 (61/66)	
Testicular volume					
Left (ml)	21.3 ± 4.4	18.5 ± 4.0	21.7 ± 4.4	20.0 ± 4.2	0.067†
Right (ml)	22.2 ± 3.6	19.7 ± 4.1	22.6 ± 3.5	20.6 ± 4.0	0.082†
Prostatic enlargement, %					
Yes	11.1 (7/63)	9.1 (5/55)	12.5 (6/48)	12.1 (8/66)	0.644*
No	88.9 (58/63)	90.9(50/55)	87.5 (42/48)	87.9 (58/66)	
History of chronic prostatitis, %					
Yes	6.4 (4/63)	12.7 (7/55)	12.5 (6/48)	10.6 (7/66)	0.067*
No	93.7 (59/63)	87.3(48/55)	87.5 (42/48)	89.4 (59/66)	
History of seminal vesiculitis, %					
Yes	3.2 (2/63)	1.8 (1/55)	4.2 (2/48)	3.0 (2/66)	0.954*
No	96.8 (61/63)	98.2 (54/55)	95.8 (46/48)	97.0 (64/66)	
Hematospermia, %					
Yes	3.2 (2/63)	1.8 (1/55)	2.1 (1/48)	3.0 (2/66)	0.957*
No	96.8 (61/63)	98.2 (54/55)	97.9 (47/48)	97.0 (64/66)	

Data are presented as mean ± SD or as median (minimum, maximum) or as percentages (%)

Abbreviations: *PA-LC* Prodom-assisted L-carnitine treatment, *PA-CoQ10* Prodom-assisted Coenzyme Q10 treatment, *PA-LC + CoQ10* Prodom-assisted L-carnitine combined with Coenzyme Q10 treatment, *OR-LC + CoQ10* oral L-carnitine combined with Coenzyme Q10 treatment, *NS* not-significant, *SD* standard deviation, *ml* milliliter, *l* liter, *pH* acidity index

*P-*values were calculated using †one-way analysis of variance (one-way ANOVA), *Chi-squared

The boldface represents statistical significance

### Analysis of the improvements to semen quality through the addition of LC/CoQ10 to semen in vitro in ASZ patients

Adding LC/CoQ10 in vitro to semen from ASZ patients is shown in Table 2. In the LC + CoQ10, LC, CoQ10 and blank control groups, there was a statistically significant difference in progressive sperm motility (*F* = 70.77, *P < 0.001)*, *and* sperm motility (*F* = 269.29, *P < 0.001)*. Multiple comparisons between groups showed that the LC + CoQ10 group had significant differences from the LC, CoQ10 and blank control groups (all *P < 0.05), and* the improvement in both the progressive and total sperm motility in the LC + CoQ10 group was higher than in the other groups. However, there was no significant difference in semen pH or volume, sperm concentration, total sperm number or normal sperm morphology between groups after the addition of the solutes (all *P > 0.05)*.

**Table 2:** Analysis of semen quality improvement by L-carnitine combined with Coenzyme Q10 supplementation in vitro semen of asthenozoospermia patients for an actual value at the end of the study

Groups Parameters	LC (*n* = 120)	CoQ10 (*n* = 120)	LC + CoQ10 (*n* = 120)	Blank control (*n* = 120)	*F/χ*^*2*^	*P*-value
Patient (Male) Age (Year)	31 (21, 47)	31 (21, 47)	31 (21, 47)	31 (21, 47)	0	1
The initial concentration of the added drug (mmol/l)	8	4	8,4	–	–	–
The initial volume of the added drug (ml)	2	2	1,1	–	–	–
The final concentration of added drug (mmol/l)	5.3 ± 0.4	2.7 ± 0.2	2.7 ± 0.2, 1.3 ± 0.1	–	–	–
Semen parameters (contains additives)						
Incubation time (Minute)	60	60	60	60	0	1
Incubation temperature (°C)	37	37	37	37	0	1
Semen volume (ml)	3.0 ± 0.2	3.0 ± 0.2	3.0 ± 0.2	3.0 ± 0.2	0	1
pH	7.4 ± 0.1	7.4 ± 0.1	7.4 ± 0.1	7.4 ± 0.1	0.25	0.862
Sperm concentration (10^6^/ml)	15.6 ± 4.2	15.8 ± 4.1	16.5 ± 4.2	16.1 ± 3.8	0.994	0.395
Total sperm number (10^6^)	48.3 ± 14.2	50.1 ± 14.5	47.6 ± 14.6	49.0 ± 13.5	0.658	0.578
Sperm morphology, %						
Normal	11 (6, 15)	10 (5, 15)	11 (6, 15)	10 (6, 14)	0.033	0.804
Progressive sperm motility, %	24.3 ± 4.6^b^	23.0 ± 4.8^a^	28.4 ± 5.0^c,d,e^	19.7 ± 4.4	70.77	**< 0.001**
Sperm motility, %	38.1 ± 5.1^b^	36.9 ± 4.4^a^	43.8 ± 5.4^c,d,e^	26.0 ± 4.9	269.29	**< 0.001**

Data are presented as mean ± SD or as median (minimum, maximum)

*P*-values were calculated using one-way analysis of variance (one-way ANOVA), and the Bonferroni t test was used for multiple comparisons. Among them, the effect of LC + CoQ10 on protects progressive sperm motility and sperm motility was different from that of LC, CoQ10 and blank control (all *P* < 0.05). It can be considered that the LC + CoQ10 is superior to the LC, CoQ10 in protecting sperm motility and live sperm rate

The boldface represents statistical significance

Abbreviations: *LC* L-carnitine, *CoQ10* Coenzyme Q10, *LC + CoQ10* L-carnitine combined with Coenzyme Q10, *SD* standard deviation, *ml* milliliter, *l* liter, *pH* acidity index

^a^Represents a significant difference between the CoQ10 group and the Blank control group

^b^represents a significant difference between the LC group and the Blank control group

^c^Represents a significant difference between the LC + CoQ10 group and the Blank control group

^d^Represents a significant difference between the LC + CoQ10 group and the CoQ10 group

^e^represents a significant difference between the LC + CoQ10 group and the LC group

### Pregnancy rate, time to conception, and treatment costs in the PA-LC + CoQ10, PA-LC, PA-CoQ10 and OR-LC + CoQ10 groups

The clinically therapeutic effects of LC and CoQ10 treated by different administration routes and different combinations are shown in Table 3. In the PA-LC + CoQ10, PA-LC, PA-CoQ10 and OR-LC + CoQ10 treatment groups, The four treatment methods were not the same in terms of the influence of pregnancy rate (***χ***^*2*^ = 3.684, *P = 0.013)*, time to conception (*F* = 99.925, *P < 0.001)*, and treatment cost (*F* = 59.932, *P < 0.001),* but the difference in miscarriage rate (***χ***^*2*^ = 0.341, *P = 0.796)* was not significant. And multiple comparisons were made between groups, the results showed that the PA-LC + CoQ10 treatment group had significant differences from the PA-LC, PA-CoQ10 and OR-LC + CoQ10 treatment groups (all *P < 0.05)*. *and* the pregnancy rate was high, the time to conception was shortened, and the cost of treatment was reduced in the PA-LC + CoQ10 group compared to the other groups.

**Table 3:** Pregnancy rate/time to conception/treatment cost between groups were compared in the patients of asthenozoospermia

Groups Parameters	PA-LC + CoQ10 (*n* = 63)	PA-LC (*n* = 55)	PA-CoQ10 (*n* = 48)	OR-LC + CoQ10 (*n* = 66)	Total (*n* = 232)	*F/χ*^*2*^	*P*-value
Pregnancy rate (%)	57.1^c,d,e^ (36/63)	38.2^b^ (21/55)	35.4^a^ (17/48)	30.3 (20/66)	40.5 (94/232)	3.684	0.013*
Abortion rate (%)	5.6 (2/36)	4.8 (1/21)	11.8 (2/17)	10.0 (2/20)	7.5 (7/94)	0.341	0.796*
Time to conception (months)	3.4 ± 0.9^c,d,e^	6.1 ± 1.8^b^	6.2 ± 1.8^a^	7.9 ± 2.0	5.5 ± 2.4	99.925	< **0.001**†
Treatment cost (US$)	1348 ± 411^c,d,e^	2350 ± 457^b^	2455 ± 434^a^	2684 ± 334	2056 ± 701	59.932	< **0.001**†

Data are presented as mean ± SD or as percentages (%)

*P*-values were calculated using †one-way analysis of variance (one-way ANOVA), and the Bonferroni t test was used for multiple comparisons, *Chi-squared test of rows x columns, and the Bonferroni correction was used for multiple comparisons. Among them, the effect of PA-LC + CoQ10 on increasing the pregnancy rate, shortening the time to conception and reducing treatment costs was different from that of PA-LC, PA-CoQ10 and OR-LC + CoQ10 (all *P* < 0.05). It can be considered that the PA-LC + CoQ10 is superior to the PA-LC, PA-CoQ10 in increasing the pregnancy rate, shortening the time to conception and reducing treatment costs

The boldface represents statistical significance

Abbreviations: *PA-LC* Prodom-assisted L-carnitine treatment, *PA-CoQ10* Prodom-assisted Coenzyme Q10 treatment, *PA-LC + CoQ10* Prodom-assisted L-carnitine combined with Coenzyme Q10 treatment, *OR-LC + CoQ10* L-carnitine combined with Coenzyme Q10 oral treatment, *SD* standard deviation

^a^Represents a significant difference between the PA-CoQ10 group and the OR-LC + CoQ10 group

^b^represents a significant difference between the PA-LC group and the OR-LC + CoQ10 group

^c^Represents a significant difference between the PA-LC + CoQ10 group and the OR-LC + CoQ10 group

^d^Represents a significant difference between the PA-LC + CoQ10 group and the PA-CoQ10 group

^e^represents a significant difference between the PA-LC + CoQ10 group and the PA-LC group

## Discussion

The present study evaluated the improvement of sperm quality in semen supplemented with LC and CoQ10 and the resulting pregnancy rates in patients with ASZ. The results showed that the addition of LC combined with CoQ10 in vitro to semen was helpful in improving both progressive sperm motility and sperm motility, and the effect was better than that of either LC or CoQ10 administered alone. Moreover, it can also improve the pregnancy rate among couples with ASZ by supplementing semen with LC and CoQ10 and injecting it into the partner’s vagina via a Prodom during sexual intercourse along with ejaculation. Similarly, the effect of using LC in combination with CoQ10 is superior to that of using LC or CoQ10 alone.

Previous literature has reported that oral LC therapy can improve progressive sperm motility to 20.1 ± 8.8% and sperm motility to 38.3 ± 9.7%, and the pregnancy rate of couples with ASZ was 19.6% [7, 17]. In this study, supplementation with LC in in vitro semen improved progressive and total sperm motility to 24.3 ± 4.6% and sperm motility to 38.1 ± 5.1%. The pregnancy rate improved to 38.2% through assistance by the device of Prodom [23, 24] supplementation of LC into the partner’s vagina during sexual intercourse and synchronized with ejaculation. This suggests that in vitro supplementation with LC is effective in the treatment of ASZ and that it is superior to in vivo supplementation [12, 26].

The literature has also shown that oral CoQ10 therapy can improve progressive sperm motility to 28.9 ± 14.8% and sperm motility to 39.4 ± 6.8% resulting in improvement of the pregnancy rate among couples with ASZ to 12.7% [7, 17]. In this study, supplementation with CoQ10 in semen in vitro improved progressive sperm motility to 23.0 ± 4.8% and sperm motility to 36.9 ± 4.4%. Thus, the pregnancy rate improved to 35.4% through assisted by the device of Prodom [23, 24] and supplementation of CoQ10 into the partner’s vagina during sexual intercourse and synchronized with ejaculation. This suggests that in vitro supplementation with CoQ10 is effective in the treatment of ASZ and that it is superior to in vivo supplementation [27, 28].

The literature also shows that oral LC combined with CoQ10 treatment can improve total and progressive sperm motility to 28.3 ± 14.1% and sperm motility to 38.8 ± 15.6% and improve the pregnancy rate among couples with ASZ to 30.0% [7, 17]. In this study, adding L-carnitine combined with CoQ10 to semen in vitro improved sperm motility by 28.4 ± 5.0% and sperm motility by 43.8 ± 5.4%. A Prodom [23, 24], a device used to administer therapeutic drugs during intercourse by adding LC and CoQ10 to semen as it enters the partner’s vagina, facilitates synchronization with ejaculation and achieved a high pregnancy rate in clinical efficacy trials. These results suggest that LC combined with CoQ10 was more effective in the treatment of ASZ in vitro than in vivo [29–31].

In this study, our results showed that the combination of LC and CoQ10 in the treatment of patients with ASZ achieved good efficacy, considering the following reasons: first, the combination of the two drugs has a complementary effect; second, supplementation directly in semen avoids the complex mechanism of metabolism in vivo and many interference factors [23, 24]; and third, a suitable external environment, especially in the vagina of a woman who is ovulating, provides a good environment for spermatozoa to obtain energy and damage protection. Further research is needed to confirm our hypothesis that sperm gain energy and are protected from damage in the vagina of ovulating women.

Prodom, an auxiliary device, is used in synchronization with ejaculation during intercourse. Previous studies [23, 24] have shed light on how it can assist chymotrypsin and urokinase release into semen in the treatment of male infertility caused by impaired semen liquefaction. The device is simple, easy to operation, and result in less pain than syringe vaginal injection therapy. In addition, the device makes it possible to mix drugs directly with semen and prevent vaginal leakage [23, 24].

In this study, we also evaluated the treatment cost, time to pregnancy, and miscarriage rates. In the PA-LC + CoQ10 group, the treatment cost was reduced, and the time to conception was shortened comparing with the PA-LC, PA-CoQ10 and OR-LC + CoQ10 groups, and the effect of PA-LC + CoQ10 was superior to that of PA-LC, PA-CoQ10 and OR-LC + CoQ10; neither the time to conception nor the cost of treating treating infertility caused by ASZ have ever been reported in the literature. We believe that our research provides a reference value. As for the occurrence of adverse events in abortion, the miscarriage rate was 5.6% (2/36) in the PA-LC + CoQ10 group, and there was no significant difference compared with the other groups. A review of previous literature reported that the miscarriage rate was between 1 and 13% [7, 17, 27], and our results did not show a significant increase. This suggests that in vitro supplementation of semen with LC and CoQ10 does not increase the risk of miscarriage.

However, the present study had several limitations. This study was based on only single-center clinical data and a relatively small number of cases. These limitations may have influenced the results and conclusions. Hence, larger and more centralized case studies are necessary. In addition, further research is needed to confirm our hypothesis that sperm gain energy and are protected from damage in the vagina of ovulating women.

## Conclusion

The results of the current study indicate that supplementation of semen with LC combined with CoQ10 in vitro can improve sperm motility. Moreover, Prodom assists in the administration of LC combined with CoQ10 to the spouse’s vagina, which increases the rate of pregnancy, shortens the time to conception and reduces the cost of treatment in patients with ASZ.

## Data Availability

The datasets generated and/or analyzed during the current study are available from the corresponding author upon reasonable request.

## Abbreviations

ASZ: Asthenozoospermia
CoQ10: Coenzyme Q10
Co., LTD.: Company limited
DNA: Deoxyribonucleic acid
l: Liter
LC: L-carnitine
OR-LC + CoQ10: Oral L-carnitine combined with Coenzyme Q10 treatment group
ml: Milliliter
PA-LC: Prodom-assisted L-carnitine treatment group
PA-CoQ10: Prodom-assisted Coenzyme Q10 treatment group
PA-LC + CoQ10: Prodom-assisted L-carnitine combined with Coenzyme Q10 treatment group
pH: Acidity index
SD: Standard deviation
